# Does journal club membership improve research evidence uptake in different allied health disciplines: a pre-post study

**DOI:** 10.1186/1756-0500-5-588

**Published:** 2012-10-29

**Authors:** Lucylynn M Lizarondo, Karen Grimmer-Somers, Saravana Kumar, Alan Crockett

**Affiliations:** 1 International Centre for Allied Health Evidence, University of South Australia, North Terrace, Adelaide, 5000, Australia

**Keywords:** Journal club, Evidence-based practice, Allied health, Evidence uptake

## Abstract

**Background:**

Although allied health is considered to be one 'unit' of healthcare providers, it comprises a range of disciplines which have different training and ways of thinking, and different tasks and methods of patient care. Very few empirical studies on evidence-based practice (EBP) have directly compared allied health professionals. The objective of this study was to examine the impact of a structured model of journal club (JC), known as *i*CAHE (International Centre for Allied Health Evidence) JC, on the EBP knowledge, skills and behaviour of the different allied health disciplines.

**Methods:**

A pilot, pre-post study design using maximum variation sampling was undertaken. Recruitment was conducted in groups and practitioners such as physiotherapists, occupational therapists, speech pathologists, social workers, psychologists, nutritionists/dieticians and podiatrists were invited to participate. All participating groups received the *i*CAHE JC for six months. Quantitative data using the Adapted Fresno Test (McCluskey & Bishop) and Evidence-based Practice Questionnaire (Upton & Upton) were collected prior to the implementation of the JC, with follow-up measurements six months later. Mean percentage change and confidence intervals were calculated to compare baseline and post JC scores for all outcome measures.

**Results:**

The results of this study demonstrate variability in EBP outcomes across disciplines after receiving the *i*CAHE JC. Only physiotherapists showed statistically significant improvements in all outcomes; speech pathologists and occupational therapists demonstrated a statistically significant increase in knowledge but not for attitude and evidence uptake; social workers and dieticians/nutritionists showed statistically significant positive changes in their knowledge, and evidence uptake but not for attitude.

**Conclusions:**

There is evidence to suggest that a JC such as the *i*CAHE model is an effective method for improving the EBP knowledge and skills of allied health practitioners. It may be used as a single intervention to facilitate evidence uptake in some allied health disciplines but may need to be integrated with other strategies to influence practice behaviour in other practitioners. An in-depth analysis of other factors (e.g. individual, contextual, organisational), or the relative contribution of these variables is required to better understand the determinants of evidence uptake in allied health.

## Background

This paper presents the findings of a pre-post study which examined the impact of a structured model of journal club on the knowledge, skills and behaviour of allied health practitioners relevant to evidence-based practice (EBP).

### Allied health perspectives on uptake of research evidence into practice

The literature suggests that allied health practitioners (AHPs) in general have positive attitudes toward EBP, and believe their clinical decisions should be supported by research evidence [1-3]. However, despite their recognition of its importance and value, the uptake of research evidence in clinical practice remains limited [1,4,5]. For example, a survey of paediatric occupational therapists and physiotherapists revealed wide variations and gaps between their actual practice and best practice guidelines in the treatment of cerebral palsy [6]. In another study which examined the current practices of occupational therapists, physiotherapists and speech pathologists, best practices in post-stroke rehabilitation were not routinely applied [7]. For many AHPs, the move towards regularly utilising evidence in practice is still an ongoing challenge.

Previous research outlines differences between and within allied health disciplines in terms of their knowledge and skills relevant to EBP [8]. Their learning needs vary according to their profession and prior research experience [9,10]. There are also considerable differences in terms of access to evidence sources and perceived support from the organisation/institution [9,11]. This body of evidence suggests that there is no ‘one-size-fits-all’ strategy that is likely to be effective across all disciplines. There needs to be recognition of the differences within and between allied health practices which require different approaches in order to influence practice behaviour.

### Journal club as a medium to bridge the gap between research and practice

A journal club (JC) is a group of individuals who regularly meet to discuss current articles from scientific journals [12]. There is evidence to suggest that JCs are one approach which can be used to bridge the gap between research and clinical practice [13-17]. They can be used to provide structured time for reading and overcome difficulties associated with understanding research findings, which have both been reported as barriers to implementing evidence into practice [15,18].

Journal clubs have been reported in different health care settings but mostly for medical and nursing professions [12,19,20]. In medicine, the literature reports significant improvements not only in JC participants’ reading habits [12,21,22] but also in their knowledge of biostatistics, research design and critical appraisal [23-27]. In nursing, on the other hand, JC participation led to improvements in critical appraisal skills of members, and better social networking among staff [28-31]. There is little information about the effectiveness of JCs in allied health.

### An innovative model of journal club –iCAHE journal club

In traditional models of JC, the presenter randomly selects an article for discussion and meetings generally consist of summarising the article based on the author’s results and conclusions [32]. Furthermore, most presenters do not examine the quality of the articles because of lack of skills in critical appraisal. As a result, the information obtained from the article is rarely reflected upon and any learnings are seldom processed for clinical use [32]. To address these issues, a structured, innovative model of JC was developed by the International Centre for Allied Health Evidence (*i*CAHE). A detailed description of the *i*CAHE model of JC, including its development and structure, has been reported elsewhere [33]. Briefly, the *i*CAHE JC aims to provide a sustainable model of JC to keep AHPs informed of the current best evidence and ultimately promote research evidence uptake. This model is based on the principles of Adult Learning or Andragogical Theory and integral to it is the nomination of facilitators who will act as the point of contact between *i*CAHE and AHPs from each JC. The *i*CAHE JC utilises a collaborative approach, where researchers from *i*CAHE and AHPs from JCs share responsibilities, as shown in Table 1. The current format of the *i*CAHE JC provides a standardised structure for conducting JCs, and the collaboration between researchers and practitioners from JCs address issues associated with lack of skills in appraisal, which makes it preferable over traditional models of JCs [34].

**Table 1:** **Summary of tasks allocated to journal clubs and *i*CAHE**

**Assigned group**	**Tasks**
Journal club	Development of clinical scenario
Journal club	Development of an answerable clinical question using the PICO or PECOT framework
	*PICO (Population, Intervention, Comparison, Outcome)
	*PECOT (Population, Exposure, Comparison, Outcome, Timeframe)
*i*CAHE	Development of appropriate search strategy
Shared responsibility of journal club and *i*CAHE	Identification, appraisal and summary of appropriate best available evidence
*i*CAHE	Publication, summary and critical appraisal provided to journal club
Journal club	Publication, summary and critical appraisal presented to journal club by facilitator and presenter

The objective of this study was to examine the impact of the *i*CAHE JC on the EBP knowledge, skills and behaviour of the different allied health disciplines.

## Methods

### Ethics

This study was approved by the University of South Australia Human Research Ethics Committee and the Human Research Ethics Committee (Tasmania) Network. Written informed consent was obtained from all participants.

### Research design

A single arm, pre-post study design, combining quantitative and qualitative approaches, was used in the study. Only the quantitative findings are reported in this paper.

### Sampling and participants

As this was a pilot study, no formal sample calculation was performed. Sampling however, aimed for maximum variation in the JCs so that a diverse range of AHPs can be studied. The objective of maximum variation sampling is to select a sample that is more representative than a random sample when a small number of participants are to be selected, which was the case in the current study [35]. Practitioners who belong to the category of allied health therapy [36] were invited to participate, including physiotherapists, occupational therapists, speech pathologists, social workers, psychologists, nutritionists/dieticians and podiatrists. To avoid sample contamination, recruitment was undertaken mainly in Tasmania, Australia where *i*CAHE had not established any JCs. Recruitment of participants was undertaken in groups rather than as individuals and therefore allied health managers were approached for nomination of groups to participate in this study. Groups were eligible to join if they satisfied the following criteria: (1) work in a health care facility in Australia, (2) agree to meet once a month for six months, and (3) have two committed facilitators. Individual practitioners were qualified to participate if they practice in Australia and were part of a group who agreed to participate in the study. Groups or individual practitioners who were previously involved in an *i*CAHE JC were excluded from the study.

### Intervention – iCAHE model of journal club

**Figure 1:**
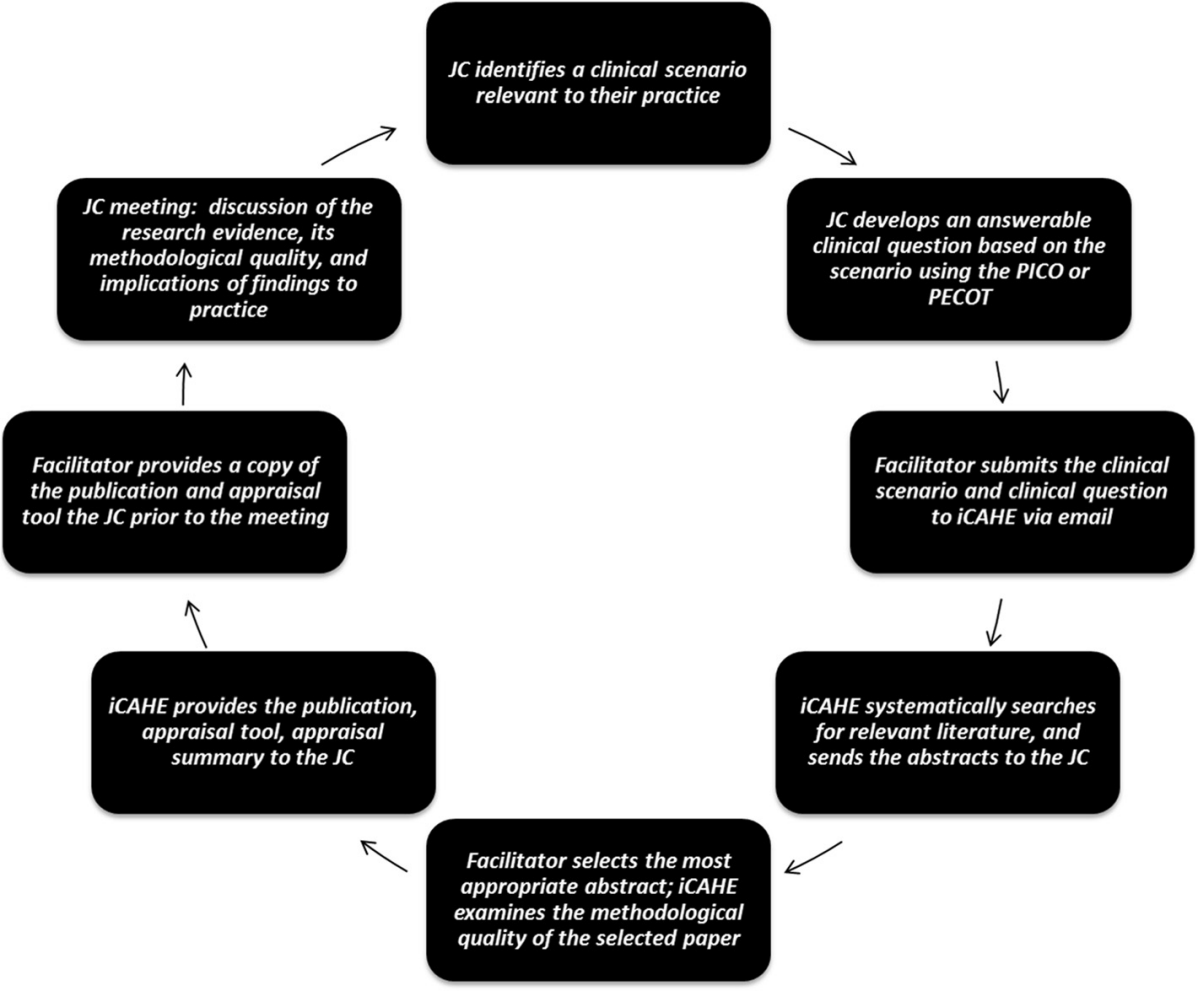
Summary of the steps involved in the intervention.

The intervention consisted of six monthly journal club sessions using the *i*CAHE model, each lasting an hour. All participating groups nominated two facilitators who were required to attend a once-off training workshop by *i*CAHE in aspects of EBP such as formulating clinical questions, developing a search strategy, critical appraisal, evidence implementation and evaluation. The facilitators were, in turn, instructed to train their members prior to the first JC session. Each round of JC involved the steps described in Figure 1.

For every meeting the facilitator led the discussion and provided members the opportunity to discuss key findings of the study, its methodological quality and issues pertaining to the implementation of research evidence to clinical practice. Self-help kits on statistics were provided by *i*CAHE when necessary. Every discussion ended with the resolution of a clinical problem and with a view towards utilising the best available evidence in making clinical decisions and evaluating its effect on practice and health care outcomes. Regular contact with *i*CAHE was maintained throughout the study.

### Data collection and analysis

Quantitative data were collected prior to the implementation of the JC, with follow-up measurements six months later. Measurements comprised the following questionnaires:

• Objective knowledge was assessed using the Adapted Fresno Test (AFT) [37]. The AFT is a seven- item instrument for assessing knowledge and skills in the major domains of EBP, such as formulating clinical questions, searching for and critically appraising research evidence. The test has acceptable validity, inter-rater reliability and internal consistency [37].

• EBP uptake was measured using the questionnaire developed by Upton and Upton [8]. EBP uptake referred to the extent to which the key steps involved in EBP (formulating a clinical question, searching for the most appropriate evidence to address the question, critically appraising the retrieved evidence, incorporating the evidence into a strategy for action, and evaluating the effects of any decisions and action taken) were integrated into day-to-day practice. This questionnaire has been reported to have adequate levels of validity and reliability [8]. In addition to EBP uptake, this questionnaire measured attitude to, and perceived knowledge about EBP.

The participants were asked to individually complete the paper and pencil version or electronic version of the questionnaires, prior to the first JC at a time convenient for them.

All analyses were performed using SAS version 9.3. An intention-to-treat analysis was applied, which regarded all non-completers as unchanged. In other words, for participants with missing post-intervention data, the baseline measurement (i.e. last observation) was carried forward as their post intervention measurement [38]. Data for baseline and post JC outcomes were presented as means and standard deviations. Mean percentage change and confidence intervals were calculated to compare baseline and post JC scores for all outcome measures. Although allied health is considered to be one 'unit' of healthcare providers for organisational purposes, it comprises a range of disciplines which have different training and ways of thinking, and different tasks and methods of patient care. Therefore the data were analysed per discipline rather than as an allied health group because the authors were interested in whether there were discipline-specific differences in responses to JC. The one-way analysis of variance (ANOVA) was used to analyse baseline differences across allied health disciplines [39,40]. A statistical test with a p value<0.05 was considered statistically significant.

## Results

### Characteristics of the sample

Of the fourteen groups of AHPs nominated by the allied health managers, only twelve groups (i.e. journal clubs) agreed to participate in the study. Heavy clinical workload was the reason provided by the two groups who did not participate. Table 2 presents the demographic characteristics of the participants. A total of 93 AHPs, including speech pathologists (SPs), physiotherapists (PTs), social workers (SWs), occupational therapists (OTs) and dieticians/nutritionists (DNs), participated in the study. The majority of participants worked full time in an acute hospital setting, held undergraduate degrees, and had more than 10 years of clinical experience. Less than half were members of professional associations.

**Table 2:** **Demographic characteristics of participants** (**N** = **93**)

**Characteristic**	**N (%)**
*Discipline*	
SP	10 (11%)
PT	19 (20%)
SW	16 (17%)
OT	36 (39%)
DN	12 (13%)
*Practice setting*	
Acute hospital	65 (70%)
Community health	25 (27%)
Not reported	3 (3%)
*Academic background*	
Undergraduate qualification	53 (57%)
Postgraduate qualification	40 (43%)
*Length of clinical practice*	
< 5 years	19 (20%)
≥ 5 but < 10 years	17 (18%)
≥ 10 years	49 (53%)
Not reported	8 (9%)
*Membership in professional associations*/*organisations*	
Yes	38 (41%)
No	53 (57%)
Not reported	2 (2%)

### Baseline scores

At baseline, there were statistically significant differences in objective knowledge (as measured by AFT) when allied health disciplines were compared (p = 0.03). The PTs showed the highest score followed by DNs, SPs, SWs and OTs. The attitude scores were also statistically different across disciplines (p = 0.02); SPs showed the highest attitude score, followed by OTs, DNs, SWs and PTs. In terms of self-reported (i.e. perceived) knowledge, scores were not statistically different across disciplines (p = 0.42). Similarly for EBP uptake, no statistically significant difference was noted when disciplines were compared (p = 0.13).

As a result of significant differences in baseline data, change in scores was interpreted as percentage change (Post-JC score – Baseline score/Baseline score x 100) from baseline. This strategy standardised change relative to the baseline scores.

Table 3 shows the pre and post JC scores and the mean percentage change per outcome measure for every discipline.

**Table 3:** **Knowledge** (**AFT** &**Self**-**reported**), **Attitude**, **EBP uptake scores of the different allied health disciplines**

**Allied health discipline**	**EBP competencies**	**Pre-test Score Mean ± SD**	**Post-test Score Mean ± SD**	**Mean Percentage Change (95% CI)**
Speech Pathology N=10	AFT Score	25.30 ± 11.94	53.80 ± 23.08	134.36 (54.80 – 213.82)*
	Self-reported knowledge	59.50 ± 11.65	65.50 ± 6.59	12.93 (2.01 – 23.85)*
	Attitude	22.50 ± 1.84	23.00 ± 2.11	2.65 (−4.16 – 9.41)
	EBP Uptake	26.20 ± 8.93	32.00 ± 6.41	42.30 (−4.76 – 89.41)
Physiotherapy N=19	AFT Score	32.26 ± 14.65	79.52 ± 18.70	245.90 (110.65 – 381.23)*
	Self-reported knowledge	51.78 ± 13.05	63.11 ± 10.98	27.35 (13.13 – 41.56)*
	Attitude	19.05 ± 3.47	21.89 ± 4.46	15.85 (6.54 – 25.26)*
	EBP Uptake	18.89 ± 6.66	26.57 ± 8.12	71.06 (12.24 – 129.88)*
Social Work N=16	AFT Score	24.19 ± 9.87	43.06 ± 14.82	141.20 (24.09 – 258.34)*
	Self-reported knowledge	58.00 ± 13.39	63.37 ± 11.03	11.28 (4.01 – 18.55)*
	Attitude	19.56 ± 3.37	20.81 ± 3.23	8.04 (−0.29 – 16.39)
	EBP Uptake	22.56 ± 8.14	26.00 ± 5.49	28.25 (4.73 – 51.77)*
Occupational Therapy N=36	AFT Score	22.75 ± 10.87	55.58 ± 22.66	198.50 (135.71 – 261.34)*
	Self-reported knowledge	55.94 ± 13.08	62.50 ± 13.93	14.27 (6.74 – 21.80)*
	Attitude	21.31 ± 3.13	21.75 ± 3.69	2.73 (−1.98 – 7.46)
	EBP Uptake	21.56 ± 7.92	22.50 ± 8.28	16.52 (−5.86 – 38.92)
Dietetics/Nutrition N=12	AFT Score	31.75 ± 9.80	59.08 ± 22.63	87.81 (50.73 – 124.93)*
	Self-reported knowledge	59.00 ± 9.91	65.92 ± 8.04	13.68 (3.39 – 23.96)*
	Attitude	21.17 ± 3.16	21.00 ± 3.38	0.20 (−8.34 – 8.75)
	EBP Uptake	18.92 ± 8.08	23.58 ± 7.22	39.18 (8.56 – 69.78)*

*: *denotes statistically significant change from baseline*, *based on CIs which did not span zero.*

### Change in scores

**Figure 2:**
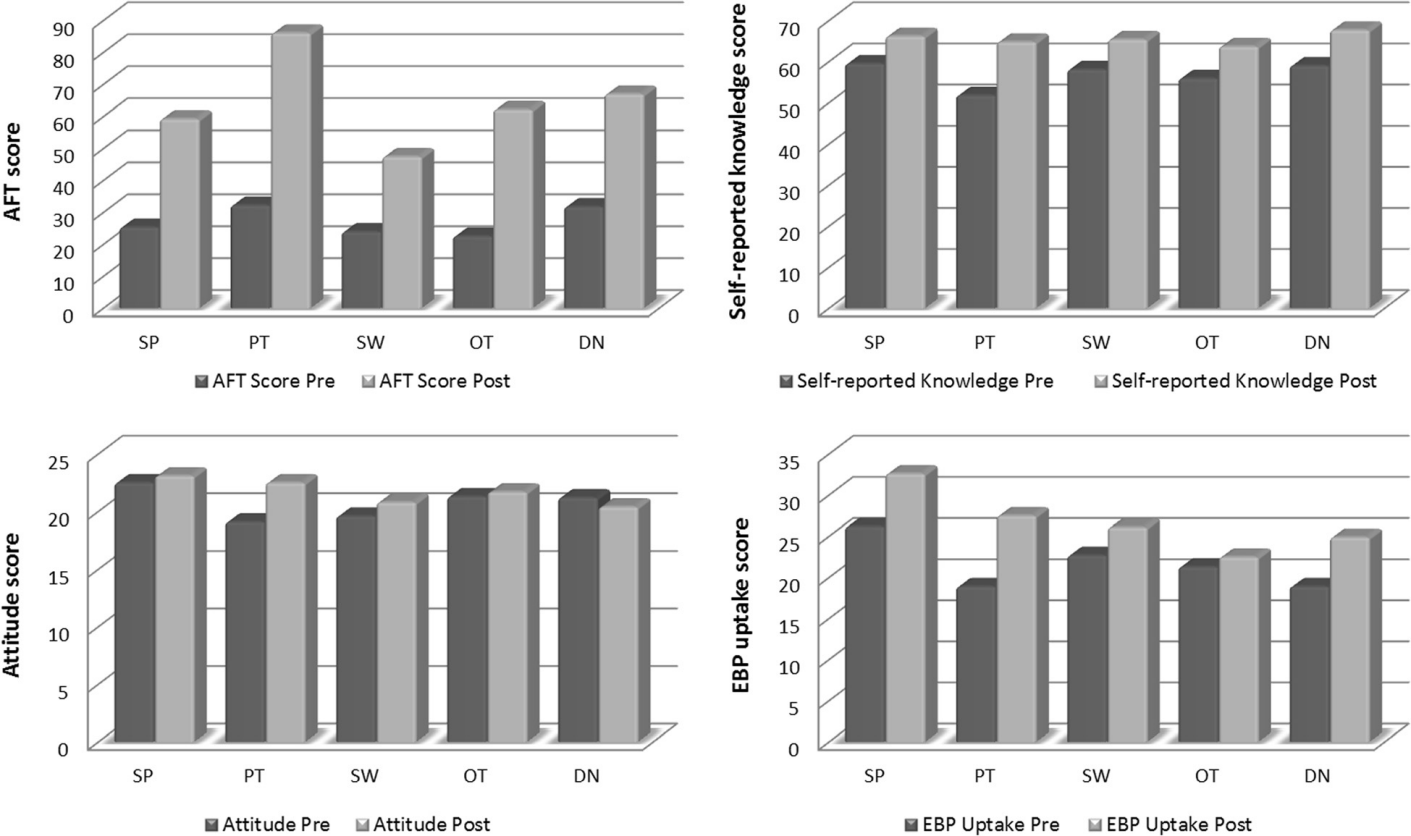
**Pre**-**post JC scores for each outcome measure in every allied health discipline.**

Figure 2 shows the pre-post scores for all outcomes in every discipline.

Following JC exposure, the AFT scores improved significantly in all disciplines. The PTs obtained the greatest change in score, followed by OTs, SWs, SPs, and DNs. There were also significant improvements in self-reported knowledge in all disciplines; greatest change was achieved by PTs, followed by OTs, DNs, SPs and SWs. No significant improvement in attitude was observed in all disciplines except for PTs. Post-JC, statistically significant improvements in EBP uptake were found for PTs, SWs and DNs but not for SPs and OTs. Table 4 summarises significant findings for all outcome measures across disciplines.

**Table 4:** Summary of significant findings for all allied health disciplines

**Allied health disciplines**	**Objective Knowledge (AFT)**	**Self-reported Knowledge**	**Attitude**	**EBP uptake**
Speech Pathology	✓	✓	×	×
Physiotherapy	✓	✓	✓	✓
Social Work	✓	✓	×	✓
Occupational Therapy	✓	✓	×	×
Dietetics/Nutrition	✓	✓	×	✓

## Discussion

The majority of EBP studies in allied health have been conducted within individual disciplines and very few empirical studies have directly compared allied health professionals. The aim of the current study was to examine the effect of an innovative model of JC – *i*CAHE model – on the EBP knowledge, attitude and behaviour of the different allied health disciplines. The results of this study demonstrated variability in EBP outcomes across disciplines after receiving the same intervention. Only the PTs showed improvements in all outcomes; SPs and OTs demonstrated an increase in both objective and perceived knowledge but not for attitude and EBP uptake; SWs and DNs showed positive changes in their objective and perceived knowledge, and EBP uptake but not for attitude. To the authors’ knowledge, this is the first study to compare EBP outcomes across allied health disciplines following an education intervention such as the *i*CAHE JC.

Based on the AFT and self-reported questionnaire, there were significant improvements in objective and perceived knowledge following exposure to *i*CAHE JC, irrespective of the discipline. This finding is consistent with the results of recent systematic reviews which showed evidence that JCs as a teaching method can increase the knowledge and confidence of health practitioners [41,42]. The literature proposes that for JCs to be successful there should be elements of adult learning principles, clearly set goals, regular meetings, use of structured critical appraisal tool, mentoring and training and distribution of journal article before the meeting [41,43,44]. The *i*CAHE model was developed in accordance with these guidelines, which could explain the satisfactory improvements in EBP knowledge and skills observed in the JC participants. There appears to be another key component in the *i*CAHE model which potentially led to the positive outcomes found in this study – the partnership between researchers and AHPs from the JCs, which was a unique feature of the *i*CAHE model. This partnership ensured that the tasks of searching, identifying and appraising relevant literature, which have all been reported as barriers to research evidence uptake, were addressed by the involvement of researchers. Therefore, the *i*CAHE model of JC did not only serve as a medium to educate AHPs with the key processes involved in EBP, but it also addressed the barriers associated with implementing evidence into practice.

This study found that AHPs vary in their attitude and behaviour outcomes to an educational intervention aimed at promoting EBP. The SPs, PTs, OTs, SWs and DNs who participated in the *i*CAHE JCs belong to the same umbrella, ‘allied health therapy.’ In 2009, a model for Australian allied health was reported by Turnbull et al., which clustered the different allied health disciplines into ‘allied health therapy’, ‘allied health diagnostic and technical’, ‘scientific services’, and ‘complementary services’. This model reflects the core tasks, training, competencies and consumer focus of the disciplines [36]. The participants of this study, even though they are grouped under the same category, responded differently to the *i*CAHE JC. The SWs and DNs showed positive changes in all EBP outcomes except for attitude while the SPs and OTs improved only in their knowledge scores. Only the PTs demonstrated significant improvements in all outcomes. There are obvious differences across allied health disciplines which can explain the variability in their outcomes. The academic and clinical training required varies considerably across professions. There are clear distinctions regarding their philosophy, scope of practice, educational standards, and competency requirements. Differences in learning styles of allied health disciplines have also been widely reported in the literature [45-47]. The research or evidence base and availability of EBP resources may also vary across disciplines [9,11]. Therefore, it is not surprising to find that while all participants were under the same classification (i.e. allied health practitioners), they showed differences in their responses to an identical intervention. The results of this study highlight the need to distinguish between disciplines, which are often treated by the EBP or research community as homogenous.

The lack of improvement in attitude following exposure to JC (except for PTs) suggests that practitioners already had positive attitude towards EBP prior to their participation in the JC, which indicates the presence of a ceiling effect. Compared to attitude, the other outcome variables showed far greater variation in scores (as shown by the larger standard deviations), which may have also played a role. Future research could explore the impact of *i*CAHE journal club in practitioners with varying levels of attitude. On the other hand, while there were a couple of disciplines (SPs and OTs) which did not show change in evidence uptake there is still reason to believe that participation in *i*CAHE JC may promote practice behaviour change. In a study by McQueen et al. (2006), findings indicated changes in practice as a result of new learning from the JC. The JC participants learned about the evidence base, and reported usage of new interventions that had been previously available but were unused due to lack of knowledge [16]. Honey and Baker (2011) reported that a JC can be used as an effective means for clinical education which can ‘*foster critical thinking about clinical practices and generate creative thinking about how practices may be carried out differently*.’ The participation in a JC emphasises the importance of critical thinking and reflective attitude in an individual practitioner, which may increase the likelihood of changing practice behaviour.

### Implications for practice

Based on the results of this study, the authors propose the use of a structured JC such as the *i*CAHE JC to improve EBP knowledge and skills in allied health. The authors believe that even though the outcomes for evidence uptake varied across disciplines, *i*CAHE JC has the potential to influence practice behaviour. However, the variability across disciplines indicates that for an EBP intervention to be effective, the strategy should be tailored to the professional discipline to facilitate and sustain an evidence-based behaviour. This could mean integrating the JC with other strategies to improve the practice behaviour of the different allied health disciplines.

### Implications for research

The authors recognise that there are other factors or variables which could have played a role in the variability of outcomes across disciplines despite receiving the same intervention. There is evidence from the literature that factors such as the characteristics of the health professional, characteristics of the organisation and contextual issues may influence evidence uptake [48,49]. Therefore, further research and an in-depth analysis of the interaction of the individual and contextual factors, or the relative importance or contribution of these variables is required to better understand the determinants of evidence uptake in allied health.

### Limitations

As with other researches, this study has limitations which need to be considered when interpreting the results. First, as this was not a controlled study, the effect of participation in other EBP-related activities or training, cannot be excluded. Second, the study did not examine the quality of the facilitation of the JC, which could have potentially affected the impact of the JC. Third, the instrument used to measure evidence uptake was a self-report questionnaire. Within the EBP literature, there is evidence to suggest that an individual’s self-assessment is often an inaccurate representation of their abilities [50,51]. The use of objective and psychometrically sound instrument to measure practice behaviour change continues to be an area which require further research. Fourth, as this was a pilot study, the findings were based on a small sample of practitioners who volunteered to participate and may not represent all AHPs. Nevertheless, the authors believe that overall, the degree of improvement demonstrated in this study lends sufficient evidence to support the *i*CAHE JC as a medium for facilitating EBP.

## Conclusions

There is evidence to suggest that a JC such as the *i*CAHE model is an effective method for improving the objective and perceived EBP knowledge and skills of AHPs. It may be used as a single intervention to facilitate evidence uptake in some allied health disciplines but may need to be integrated with other strategies to influence practice behaviour in other practitioners. The results of this study highlight the need to distinguish between disciplines and implement interventions tailored to their needs in order to achieve positive and sustainable changes in behaviour.

## Competing interests

The authors declare that they have no competing interests.

## Authors’ contributions

LML conceived of the study, collected and analysed data, and drafted the manuscript. KGS collected and analysed data and helped draft the manuscript. SK and AC participated in the design of the study and analysed data. All authors read and approved the final manuscript.
